# Supplementary material for: Synergistic Interaction of the Class IIa HDAC Inhibitor CHDI0039 with Bortezomib in Head and Neck Cancer Cells
Source: Int J Mol Sci. 2023 Mar 14;24(6):5553. doi: 10.3390/ijms24065553 (PMC10056166; doi:10.3390/ijms24065553)
Supplement: Supplementary file 1 [file ijms-24-05553-s001.zip › ijms-2237195-supplementary.pdf]

Synergistic Interaction of the Class IIa HDAC Inhibitor CHDI0039 with Bortezomib in Head and Neck Cancer Cells

Christian Schrenk [1], Lukas M. Bollmann [1], Corinna Haist [2], Artur Bister [2], Constanze Wiek [2], Maria Wecker [3], Dennis Roth [3], Patrick Petzsch [4], Karl Köhrer [4], Alexandra Hamacher [1], Helmut Hanenberg [2], Georg Fluegen [3] and Matthias U. Kassack [1,*]

1 Institute of Pharmaceutical and Medicinal Chemistry, Heinrich-Heine-University Duesseldorf, Germany
2 Department of Otorhinolaryngology, Head & Neck Surgery, Heinrich Heine University, Duesseldorf, Germany
3 Department of General, Visceral and Pediatric Surgery, Medical Faculty, University Hospital of the Heinrich-Heine-University Düsseldorf, Düsseldorf, Germany
4 Biological and Medical Research Centre (BMFZ), Genomics & Transcriptomics Laboratory, Heinrich-Heine-University Duesseldorf, Germany
* Correspondence: matthias.kassack@hhu.de

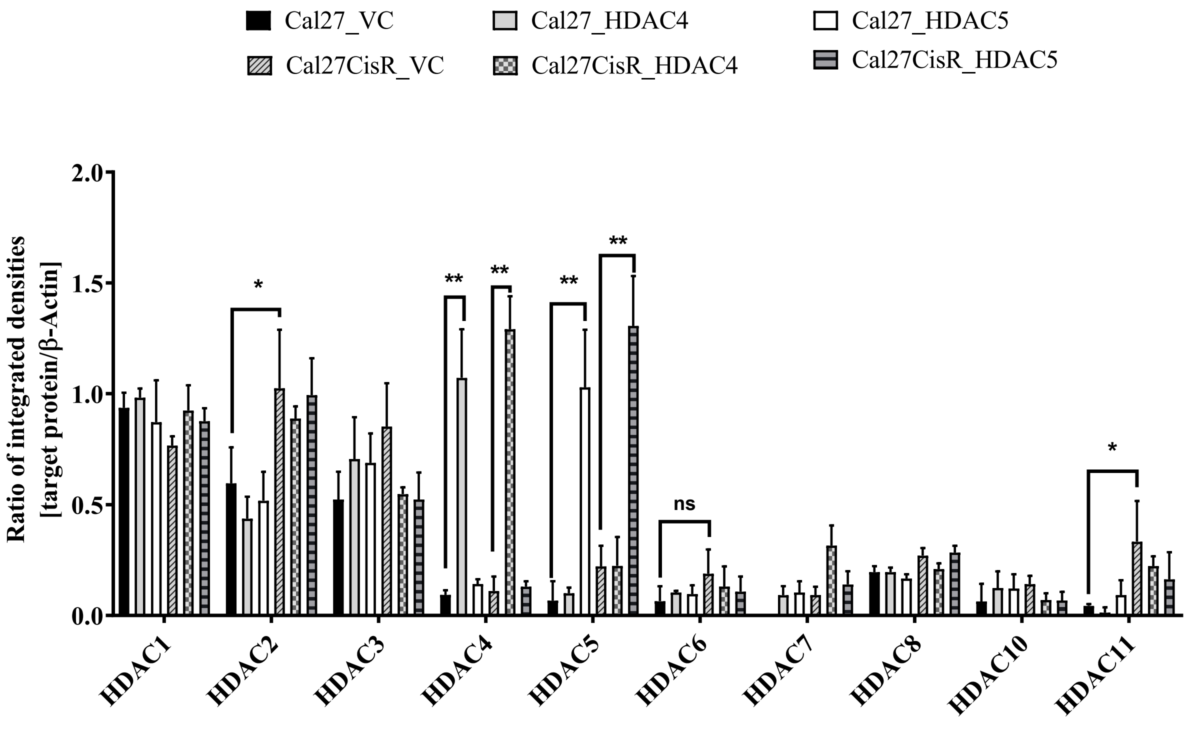

Figure S1. Quantification of western blot data from Figure 1b. Western blots were densitometrically evaluated using Image J software (Rueden, C. T., Schindelin, J., Hiner, M. C., DeZonia, B. E., Walter, A. E., Arena, E. T., & Eliceiri, K. W. ImageJ2: ImageJ for the next generation of scientific image data. *BMC Bioinformatics* **2017**, 18, doi:10.1186/s12859-017-1934-z) according to an internal protocol from H. Davarinejad (Davarinejad, H. Quantifications of Western Blots with ImageJ. Available online: www.yorku.ca/yisheng/Internal/Protocols/ImageJ.pdf, accessed on 26 February 2023). Shown are the ratios of the integrated densities of the target proteins normalized to β-actin. HDAC4 and HDAC5 overexpression clones show significant upregulation of HDAC4 and HDAC5, respectively, compared to vector control cell lines. Further, HDAC2 and HDAC11 expression is significantly increased in the

cisplatin-resistant Cal27CisR_VC compared to the sensitive Cal27_VC cells. Statistical analysis was performed using t-test. * ($p \leq 0.05$), ** ($p \leq 0.01$). Graphs represent results from at least two independent experiments (except for HDAC7 and HDAC8, only one experiment).

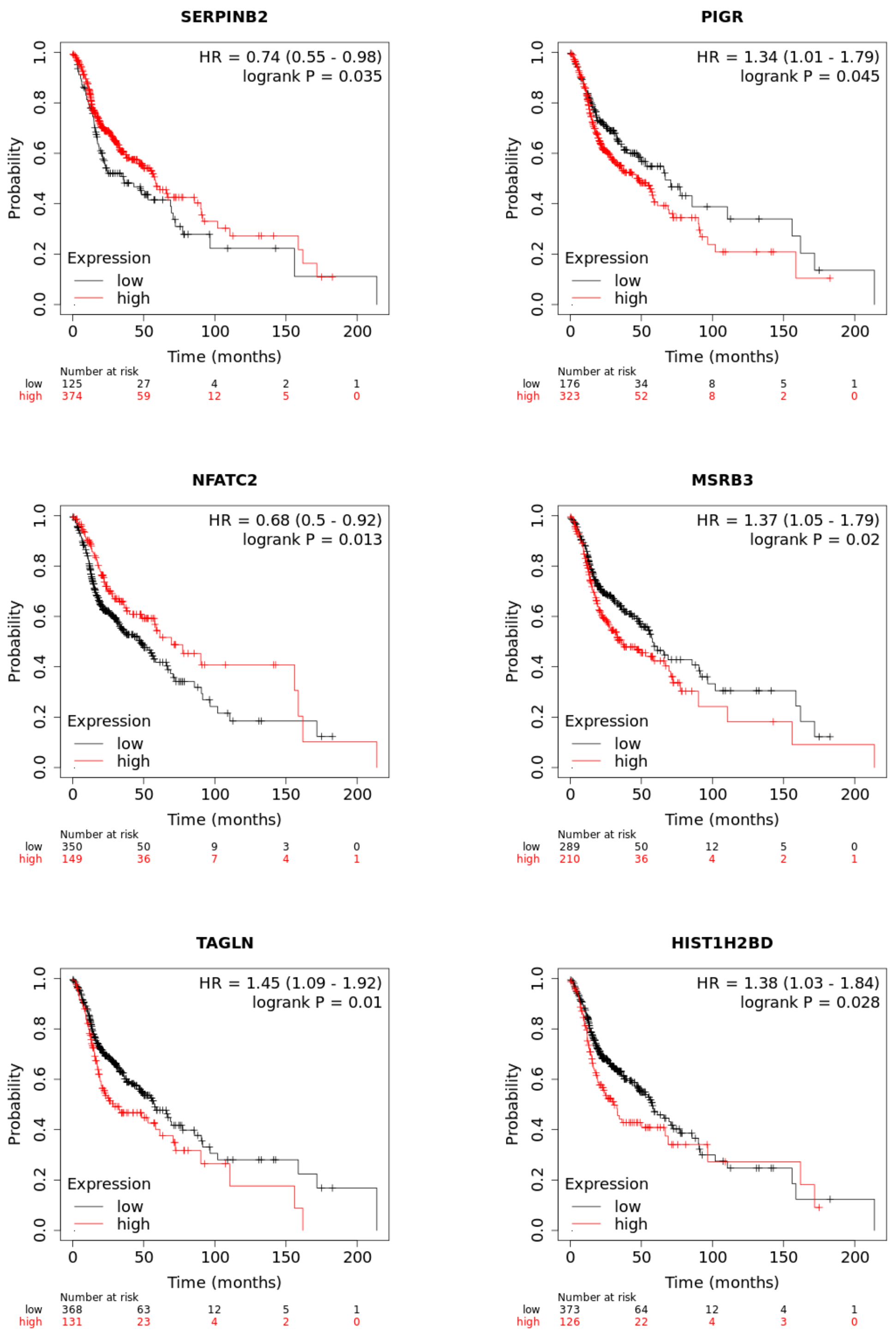

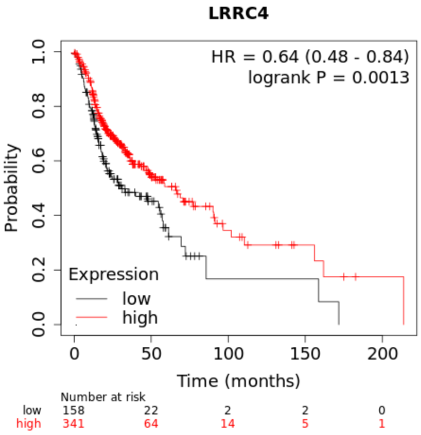
LRRC4
HR = 0.64 (0.48 - 0.84)
logrank P = 0.0013
Probability
Time (months)
Expression
low
high
Number at risk
low 158 22 2 2 0
high 341 64 14 5 1

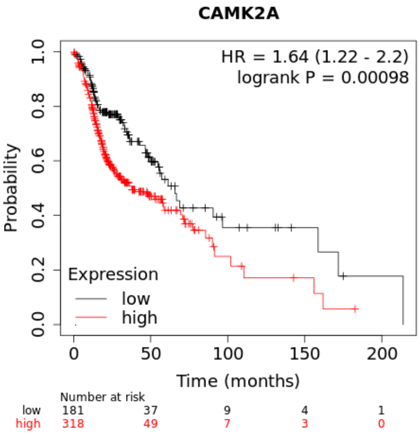
CAMK2A
HR = 1.64 (1.22 - 2.2)
logrank P = 0.00098
Probability
Time (months)
Expression
low
high
Number at risk
low 181 37 9 4 1
high 318 49 7 3 0

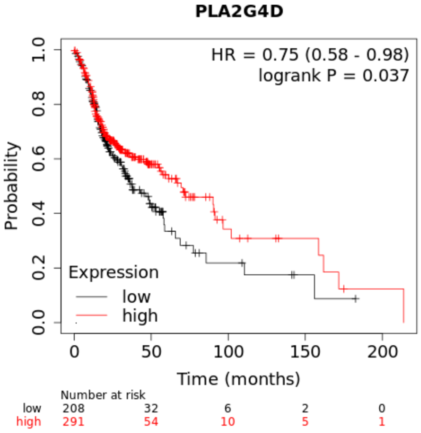
PLA2G4D
HR = 0.75 (0.58 - 0.98)
logrank P = 0.037
Probability
Time (months)
Expression
low
high
Number at risk
low 208 32 6 2 0
high 291 54 10 5 1

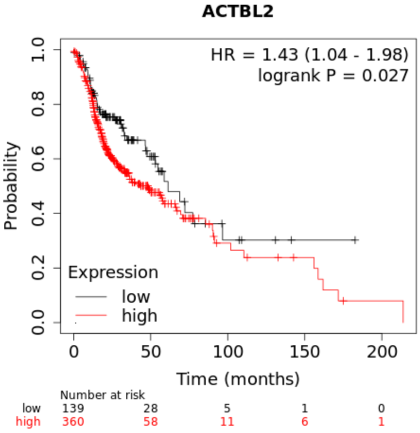
ACTBL2
HR = 1.43 (1.04 - 1.98)
logrank P = 0.027
Probability
Time (months)
Expression
low
high
Number at risk
low 139 28 5 1 0
high 360 58 11 6 1

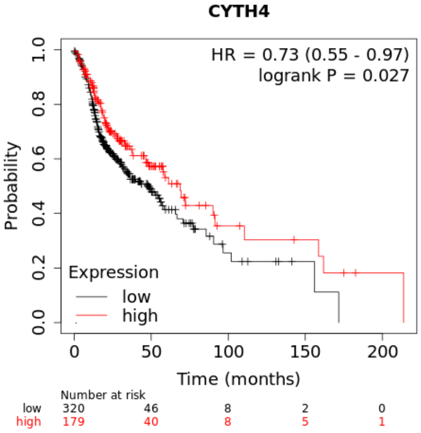
CYTH4
HR = 0.73 (0.55 - 0.97)
logrank P = 0.027
Probability
Time (months)
Expression
low
high
Number at risk
low 320 46 8 2 0
high 179 40 8 5 1

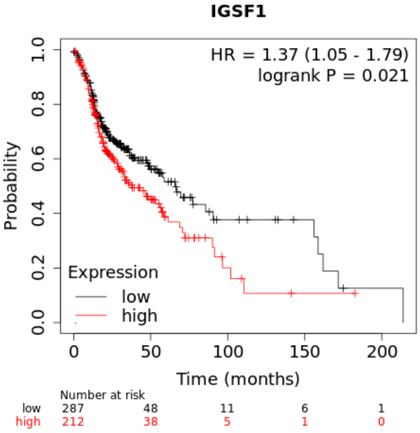
IGSF1
HR = 1.37 (1.05 - 1.79)
logrank P = 0.021
Probability
Time (months)
Expression
low
high
Number at risk
low 287 48 11 6 1
high 212 38 5 1 0

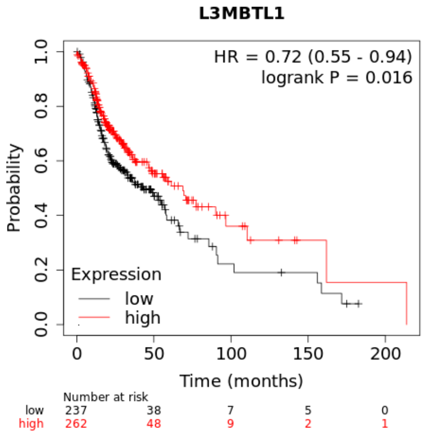

Figure S2. Kaplan Meier plots showing median survival of head and neck cancer patients with low or high expression levels of SERPINB2, PIGR, NFATC2, MSRB3, TAGLN, HIST1H2BD, LRRC4, CAMK2A, PLA2G4D, ACTBL2, CYTH4, IGSF1 and L3MBTL1. Selected genes are differently expressed between 5 μM CHDI0039-treated Cal27_HDAC4 compared to untreated Cal27_HDAC4 (RNA-seq: Bonferroni p value ≤ 0.05; fold change ≥ 2; Kaplan-Meier log rank p value ≤ 0.05). Data were extracted from publicly available database KM plotter (https://kmplot.com, accessed on 15 October 2022) described in: Lanczky, A.; Gyorffy, B. Web-Based Survival Analysis Tool Tailored for Medical Research (KMplot): Development and Implementation. *J Med Internet Res* **2021**, *23*, e27633. doi: 10.2196/27633.
